# Mutation of Vav1 adaptor region reveals a new oncogenic activation

**DOI:** 10.18632/oncotarget.2629

**Published:** 2014-10-24

**Authors:** Lyra Razanadrakoto, Françoise Cormier, Vanessa Laurienté, Elisabetta Dondi, Laura Gardano, Shulamit Katzav, Lionel Guittat, Nadine Varin-Blank

**Affiliations:** ^1^ INSERM, UMR 978, Bobigny, France; ^2^ PRES SPC, Labex Inflamex, Université Paris 13, UFR SMBH, Bobigny, France; ^3^ INSERM, UMR 1016, Institut Cochin, Paris, France; ^4^ CNRS, UMR 8104, Paris, France; ^5^ PRES SPC, Université Paris Descartes, Paris, France; ^6^ The Hebrew University/Hadassah Medical School, Jerusalem, Israel

**Keywords:** Vav1, β-catenin, Rac GTPase, Src-homology domains, adhesion complex, tumorigenesis

## Abstract

Vav family members function as remarkable scaffold proteins that exhibit both GDP/GTP exchange activity for Rho/Rac GTPases and numerous protein-protein interactions via three adaptor Src-homology domains. The exchange activity is under the unique regulation by phosphorylation of tyrosine residues hidden by intra-molecular interactions. Deletion of the autoinhibitory N-terminal region results in an oncogenic protein, onco-Vav, leading to a potent activation of Rac GTPases whereas the proto-oncogene barely leads to transformation. Substitution of conserved residues of the SH2-SH3 adaptor region in onco-Vav reverses oncogenicity. While a unique substitution D797N did not affect transformation induced by onco-Vav, we demonstrate that this single substitution leads to transformation in the Vav1 proto-oncogene highlighting the pivotal role of the adaptor region. Moreover, we identified the cell junction protein β-catenin as a new Vav1 interacting partner. We show that the oncogenicity of activated Vav1 proto-oncogene is associated with a non-degradative phosphorylation of β-catenin at residues important for its functions and its redistribution along the cell membrane in fibroblasts. In addition, a similar interaction is evidenced in epithelial lung cancer cells expressing ectopically Vav1. In these cells, Vav1 is also involved in the modulation of β-catenin phosphorylation. Altogether, our data highlight that only a single mutation in the proto-oncogene Vav1 enhances tumorigenicity.

## INTRODUCTION

The Vav1 proto-oncogene has a restricted hematopoietic expression and exhibits both GTP/GDP exchange activities (GEF) for Rho family GTPases and adaptor functions within signalling complexes [1, 2]. Two other genes, Vav2 and Vav3 belong to the same family of signalling effectors and share high structural similarities and properties with Vav1. Unlike Vav1, Vav2 and Vav3 have an ubiquitous expression [3, 4]. Vav proteins display a number of characteristic structural domains with homology for: Calponin (CH), Dbl (DH), Pleckstrin (PH) and Src (SH2 and SH3) altogether with acidic residues-rich (AcR) and cysteine-rich (CR) motives. These domains mediate interactions with membrane receptors, signalling intermediates, cytoskeleton related proteins and nuclear factors [5]. Vav proteins also exhibit a unique regulation of the GEF activity through receptor-mediated phosphorylation of tyrosine residues (pY) present in the AcR domain [6].

The initial characterization of Vav1 consisted in a truncated version of the proto-oncogene (wt-Vav1) with deletion of the first 66 residues within the CH domain that resulted in a transforming protein (onco-Vav) when expressed in fibroblasts [7]. Further deletion including the AcR motive (1-186 amino acids) or substitution of phenylalanine for the regulatory tyrosines abrogated the phosphorylation dependency of the transforming activity [8, 9]. To date, these oncogenic forms of Vav1 have never been found in neoplasms. Nevertheless, ectopic expression of Vav1 is detected and involved in the pathophysiology of neuroblastoma, pancreatic adenocarcinoma, melanoma, lung and breast cancer [10-14]. In pancreatic tumours, Vav1 regulates cell-cycle progression, proliferation, transformation and invasive migration through its interaction with dynamin 2 [10, 15]. Vav1 is also expressed in a majority of breast carcinoma where its activity is dependent on the p53 status of the cells since it induces apoptosis in wild-type p53 cells but contributes to proliferation in p53−/− cells [14]. Moreover, mutation within the N-terminal regulatory region correlates with central nervous system immune-mediated disease [16]. Critical roles have also been attributed to Vav2 and Vav3 in breast or skin cancers and in metastatic dissemination [17-19]. Altogether, these data indicate that Vav proteins display a pivotal role in tumorigenesis although not fully characterized.

The structural complexity and high similarities of Vav proteins have indicated both GTPase-dependent and GTPase-independent roles. Truncations of the inhibitory CH or AcR domains resulted in deregulated GEF activity, constitutive activation of Rho proteins and transformation [8, 20]. However, protein-protein interactions mediated by specific structural domains of Vav might also contribute to cellular transformation. In particular, the CR motif contributes to the stabilization of the DH domain and to an efficient GEF activity [21]. Intact SH2 and SH3 domains are also critical for the transforming potential of onco-Vav; mutations of conserved residues within the SH2 domain of onco-Vav affect or abrogate oncogenicity due to the loss of association with tyrosine-phosphorylated proteins [22]. Similarly, mutations of conserved residues of the C-terminal SH3 domain reversed the transforming potential of onco-Vav; while mutation of a Vav-family specific residue in the carboxyl-terminal SH3 domain (D797) was unsignificant to onco-Vav oncogenicity and maintained the binding capacity to several interacting partners [23]. This region also contributes strongly to the intramolecular inhibition of Vav family proteins [24]. Moreover, not only Vav2 and 3 but also Vav1 phosphorylation can promote Rho family activation in non-hematopoietic tissues following stimulation of several growth factor receptor tyrosine kinases [25-27]. These Vav-mediated mechanisms lead to reorganization of actin cytoskeleton and cell-cell contacts and increased motility [12, 28]. Notably, Vav2-mediated modulation of p120 catenin - E-cadherin clustering has been implicated in fibroblasts motility and in Rac-dependent activation of β catenin [29-31]. Therefore, ectopic expression of Vav1 and its capacity to interact with specific proteins in non-hematopoietic cells contribute likely to its transforming potential and to tumorigenesis.

In this study, we investigated whether alterations within the protein-protein interaction CSH3 domain might activate the transforming properties of the Vav1 proto-oncogene. We identified D797 as one novel key residue controlling Vav1 oncogenicity, since its unique mutation confers transforming properties to Vav1 proto-oncogene. We also identified β-catenin as a new Vav1 interacting partner in fibroblasts and epithelial lung cancer cells. The transformation, which is dependent on Rac activation, is associated with the phosphorylation and the stabilization of β-catenin altogether with its relocation from junctional complexes to random membrane localization in fibroblasts. Our data provide novel insights on the mechanism of Vav1-regulated oncogenesis that involves cell-cell contact dysregulation.

## RESULTS

### Mutation of the specific aspartic acid 797 residue in Vav1 C-SH3 domain activates its transforming and tumorigenic potential

**Figure 1:**
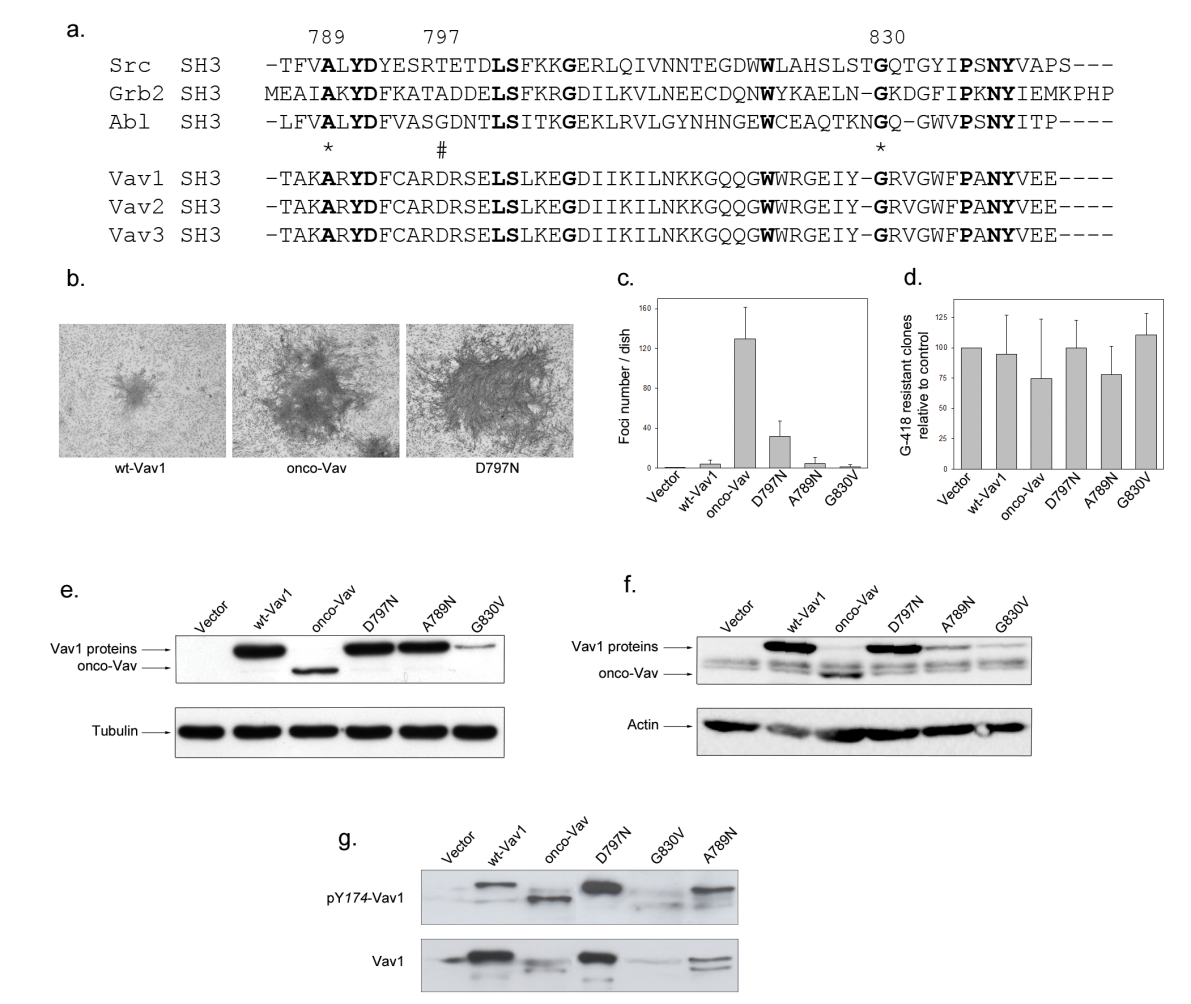
Transforming potential of the D797N Vav mutant a. Sequence alignment of SH3 domains containing proteins. SH3 domains of Src, Grb2 and Abl are used as a reference for Vav1-2-3 C-terminal SH3 domains. Conserved residues are in bold. Stars and double string indicate the mutated residues used in the study. b-c-d Focus formation assays. Two weeks after transfection with the indicated constructs, NIH3T3 cells were fixed, stained with Giemsa solution, observed using light microscopy (b, Nikon camera DXMI200F, magnification 20x) and the foci numbers were quantified (c). After selection with G-418, neo-resistant clones were counted relative to transfection with empty vector (d). Results are the mean of four independent experiments ± standard deviation. e-f. Vav1 proteins expression. Cell extracts from 48h transient transfections (e) or G418-selected stable cell lines (f) expressing the myc-tagged Vav1 proteins were analysed by immmunobloting with anti-myc Ab (upper panel). Loading controls were assessed by detection of actin and tubulin (lower panel). g Phosphorylation levels of Vav1 proteins in stable cell lines. Proteins were analysed by immmunobloting using phospho Tyr174-Vav1 antibody (upper panel) and anti-Vav1 antibody (lower panel).

The C-terminal SH3 adaptor region of Vav1 contains several conserved residues present in Src, Grb2 or Abl SH3 domains but also unique residues specific for Vav family members (Figure 1a). We analysed a selection of Vav1 proto-oncogene CSH3 mutants: D797N, A789N and G830V, for their capacity to induce foci of transformed cells when expressed in NIH3T3 cells and compared them to onco-Vav (Δ1-66). Interestingly, mutation of D797 residue activates significant transformation potential, inducing foci similar albeit to a lesser extent than Δ1-66 onco-Vav. Despite equivalent transfection efficiencies, as verified by the comparable amounts of G418-resistant clones obtained with the various constructs, none of the other mutants as well as wt-Vav1 induced transformed foci (Figure 1b, c and d). Immunoblot analysis confirmed that the transforming potential of the D797N mutant could not be ascribed to higher levels of protein expression. Two days after transfection or in established stable cell lines, D797N mutant and wt-Vav1 showed similar levels of expression and higher levels than onco-Vav that displayed the greatest transforming capacity (Figure 1e and f). Kinetic analysis of the mutated proteins in the presence of cycloheximide validated the steady state levels observed in stable cell lines showing a weaker stability for A789N or G830V when compared to D797N, onco-Vav or wt-Vav1 (Figure 1f and supplemental Figure S1). Moreover, we noticed higher levels of phosphorylated D797N and onco-Vav1 proteins compared to wt-Vav1 reflecting an increased activation of both mutants in these cell lines (Figure 1g).

**Figure 2:**
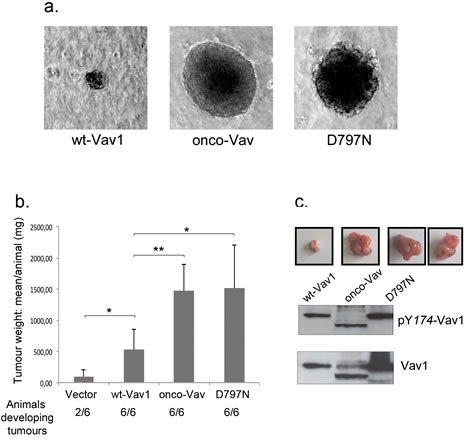
Anchorage-independent growth and tumorigenic potential of the D797N Vav1 mutant a. NIH3T3 stable cell lines were cultured in soft-agar medium as described in Methods. After 3 weeks, colonies were observed using light microscopy (Leica DMI6000, Camera MicroMAX 1300Y/HS, magnification 10x). b. The indicated NIH3T3 cells (10^6^) were injected subcutaneously in Nu/Nu mice. Three weeks after injection, tumours were harvested and weighted. Means ±SD of 4 independent experiments performed in duplicate are indicated. The number of injected animals developing tumours is also indicated. c Imaging of the tumours and phosphorylation levels of Vav1 proteins from tumour cells. Proteins were analysed by immmunobloting using phospho Tyr174-Vav1 antibody (upper panel) and anti-Vav1 antibody (lower panel). Examples of the extracted tumours are shown on top of the western blot for the 3 constructs (2 images for D797N).

Next, we compared the proliferative properties of stable cell lines expressing wt-Vav1, onco-Vav or D797N mutant. Higher proliferation rates and saturating densities were observed for transforming proteins-expressing cells while wt-Vav1-expressing cells exhibited an intermediate growth rate as compared to control with empty vector (Figure S2a). Unlike control or wt-Vav1, cells expressing onco-Vav and D797N overgrew the monolayer and proliferated beyond confluence (Figure S2a). Moreover, in reduced serum conditions, although wt-Vav1 or control cells showed poor proliferation rates, cells expressing onco-Vav and D797N lost high serum dependence (Figure S2b). Finally, loss of anchorage requirement was observed for onco-Vav or D797N expressing cells that displayed clonal expansion developed in semi-solid agar cultures (Figure 2a). In contrast, wt-Vav1 expressing cells engendered very few clones of reduced size. D797N-transformed fibroblasts also acquired the capacity to generate tumours in Nu/Nu mice after subcutaneous injection as did onco-Vav and all animals injected with these cells developed tumours. The overall weight of the tumours generated by onco-Vav and D797N-expressing cells was highly above those generated by wt-Vav1 or empty vector when present (Figure 2b). However, D797N-derived tumours showed a more heterogeneous size than did onco-Vav while wt-Vav1 expressing cells only generated small nodes (Figure 2c). Interestingly, Vav1 proteins extracted from the tumour cells exhibited activated pY174 at levels comparable to those observed in injected cells reflecting Vav1 activation (Figure 2c and 1g).

Altogether these data demonstrate that one unique CSH3 specific substitution into Vav1 proto-oncogene confers activation to the protein leading to transformation and tumorigenesis.

### Rac1 and RhoA GTPases-dependent pathways are required for D797N-induced transformation

**Figure 3:**
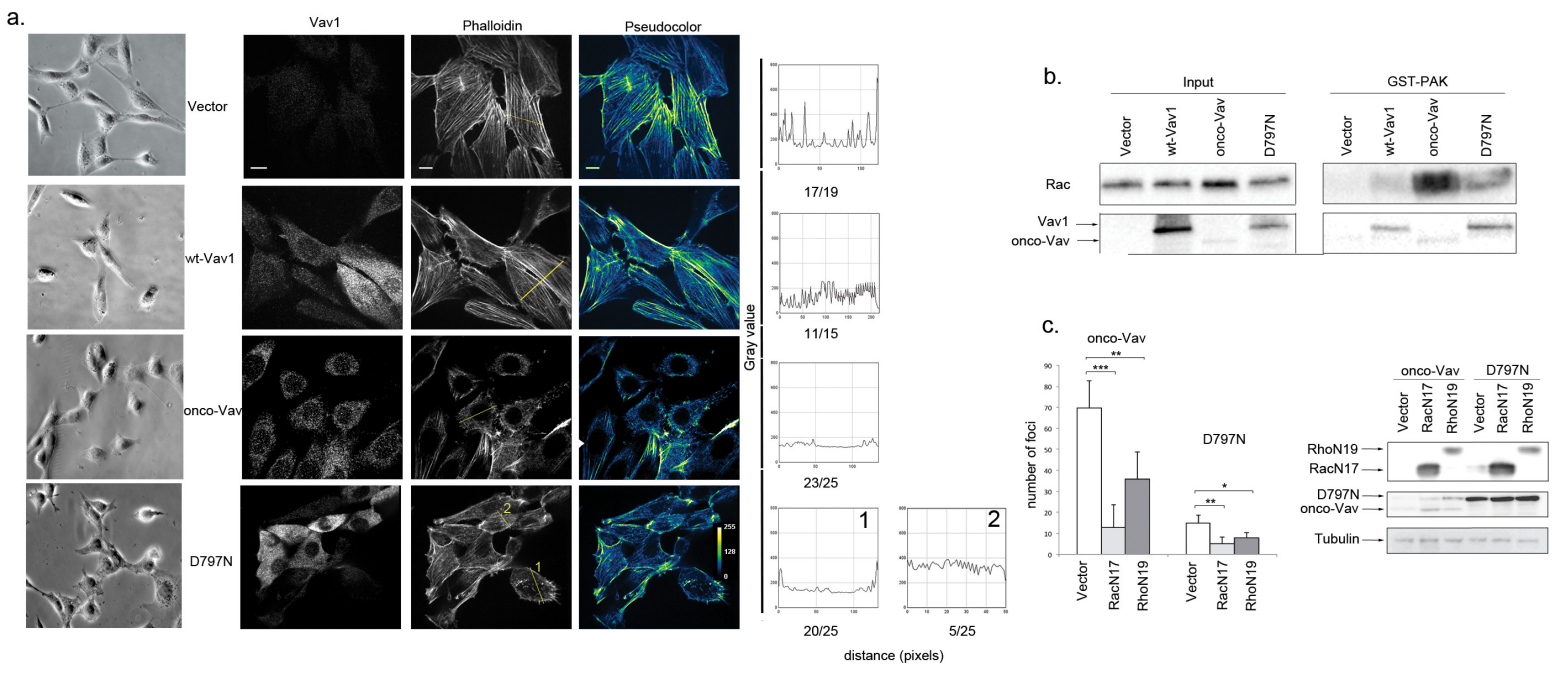
Rac1 and RhoA GTPases are effectors for D797N Vav1 mutant-induced transformation a. Cytoskeleton analysis. The indicated stable NIH3T3 cells lines were observed using light microscopy (Leica DMI6000, Camera MicroMAX 1300Y/HS, magnification 20x, first left panel) or were immunostained with Alexa 647-conjugated-phalloidin (right and middle panels) and anti-Myc Tag Ab followed by Alexa 488-conjugated anti-mouse Ig Ab (Vav1, second left panel). Pseudocolor images indicate the intensity gradients of cytoskeleton labelling (right panel). Bar = 12μm. Confocal images were acquired with a BD Pathway 855 Bioimaging System with a 40x magnification and analyzed by ImageJ software. A line was drawn across at least 15 cells from each different clone and linescan profiles of fluorescence intensity were obtained using the Analyze-Plot profile function. The numbers of cells presenting the indicated profiles are indicated for each cell line under the plot. b Rac1 activation. Rac1 activity was assessed in stable NIH3T3 cell lines using GST-Pak1 pull-down experiments (n=3). A representative experiment is shown (right panel). Cellular extracts were also analysed by immunoblottting for Rac1 and Vav1 expression (left panel) c. Rac1/RhoA are required for foci formation. NIH3T3 cells were transfected with Vav1 constructs altogether with empty, RacN17 or RhoN19 pRK5-myc based vectors as indicated. The number of foci was scored for each transfection. Columns represent the mean ± SD of 3 independent experiments performed in duplicate (left panel). Expression of Vav1, RacN17 and RhoN19 proteins was monitored by immunoblotting (right panel).

The transforming activity of all N-terminal deleted Vav oncoproteins has been ascribed to uncontrolled GEF activity on GTPases of the Rho family [6, 9, 20, 32]. Therefore, we investigated whether D797N-induced transformation might similarly depend on Rho GTPases activation. Cell morphology and cytoskeleton organization of onco-Vav- or D797N-transformed cells suggested activation of the GTPases with typical formation of ruffles (lamellipodia and filipodia) (Figure 3a; [33]). These cells also exhibited a large reduction of stress fibers that were abundantly present in control or wt-Vav1-expressing fibroblasts (Figure 3a, Pseudocolor and quantification of stress fibers and/or ruffles). Using pull-down experiments with a glutathione S-transferase (GST) fusion protein containing the Rac1 binding domain of p21protein-activated protein kinase 1 (PAK), we observed a significant activation of Rac1 GTPase in onco-Vav and D797N-expressing cells and a faint activation in wt-Vav1-expressing cells. These results also confirmed the differential transforming potential of onco-Vav and D797N (Figure 3b). Additionally, overexpression of the dominant-negative form of Rac1, RacN17, strongly repressed foci formation for both constructs. Comparable repression was obtained using the dominant-negative form of RhoA, RhoN19, albeit to a lesser extent that might be attributed to a weaker expression of the GTPase (Figure 3c).

**Figure 4:**
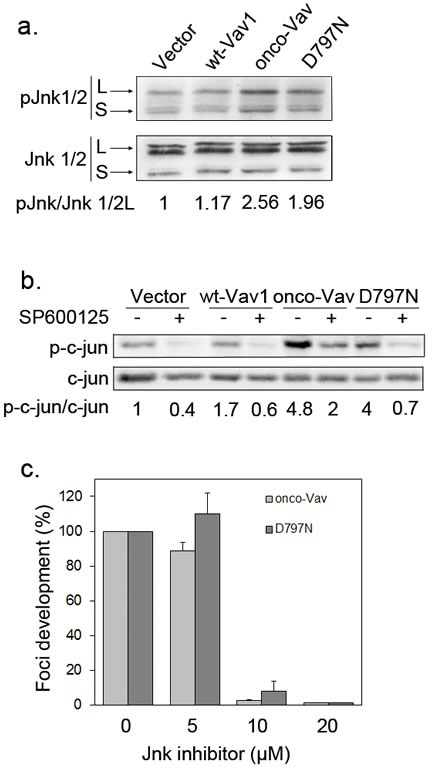
The transforming potential of D797N Vav1 implicates a JNK-dependent pathway a-b. JNK1/2 and c-Jun phosphorylation analyses. Protein extracts from stable NIH3T3 cell lines were analysed by sequential immunoblotting with (a) anti-phospho-JNK (Thr183/Tyr185) and anti-JNK Abs; (b) anti-phospho-c-Jun (Ser63) and anti-c-Jun Abs. Fold increases of phospho-JNK1/2 and phospho-c-Jun in cells expressing wt-Vav1, onco-Vav and D797N compared to control vector were normalized to whole Jnk1/2 and c-Jun expression, respectively. Inhibition of c-Jun phosphorylation was monitored after 2h treatment (+) or not (−) with the JNK inhibitor SP600125 (10μM). c. JNK activity is required for onco-Vav and D797N-induced foci formation. Focus formation assays were performed with NIH3T3 cells transfected with the indicated constructs and 0, 5, 10 or 20 μM of SP600125. Foci formation is calculated relative to untreated cells (100%). Results are means ± SD of 3 independent experiments performed in duplicate.

To explore downstream events mediating the transforming activity, we investigated the activation of c-jun NH2-terminal (JNK) and of stress-activated protein p38 (SAPK) kinases, two direct effectors of Vav and Rac/Rho GTPases [32]. Immunoblot assay, using a specific antibody for phosphorylated Thr183/Tyr 185 JNK1/2, demonstrated an enhanced activation upon expression of onco-Vav or D797N as compared to wt-Vav1 or control vector (Figure 4a). A weaker activation of p38 SAPK was also detected in the transformed fibroblasts (Figure S3a). Activation of JNK kinases yielded to the phosphorylation of c-Jun, an important target involved in cell proliferation and tumorigenesis [34]. As demonstrated by immunoblotting with anti-phospho-Ser63 c-Jun, after incubation or not with the SP600125 specific JNK inhibitor, a strong JNK-dependent activation of c-Jun was found in onco-Vav- and D797N-expressing cells, while a weaker activation was noticed for wt-Vav1 (Figure 4b).

Implication of JNK kinases in onco-Vav- and D797N-mediated transformation was further confirmed using the SP600125 specific inhibitor. As shown in Figure 4c, addition of 10μM SP600125 completely prevented foci formation by both onco-Vav- and D797N-expressing cells. In contrast, addition of 15μM SB203580, a p38 SAPK inhibitor, only reduced by two fold the number of foci (Figure S3b).

Altogether these results indicate that, similarly to onco-Vav-, D797N-mediated transformation involves the GEF activity toward Rac/Rho GTPases leading to typical cell morphology changes and to the subsequent activation, albeit to a lesser extent, of JNK-dependent pathways.

### β-catenin is a molecular target of Vav1/JNK activation and a new Vav1 interacting partner

**Figure 5:**
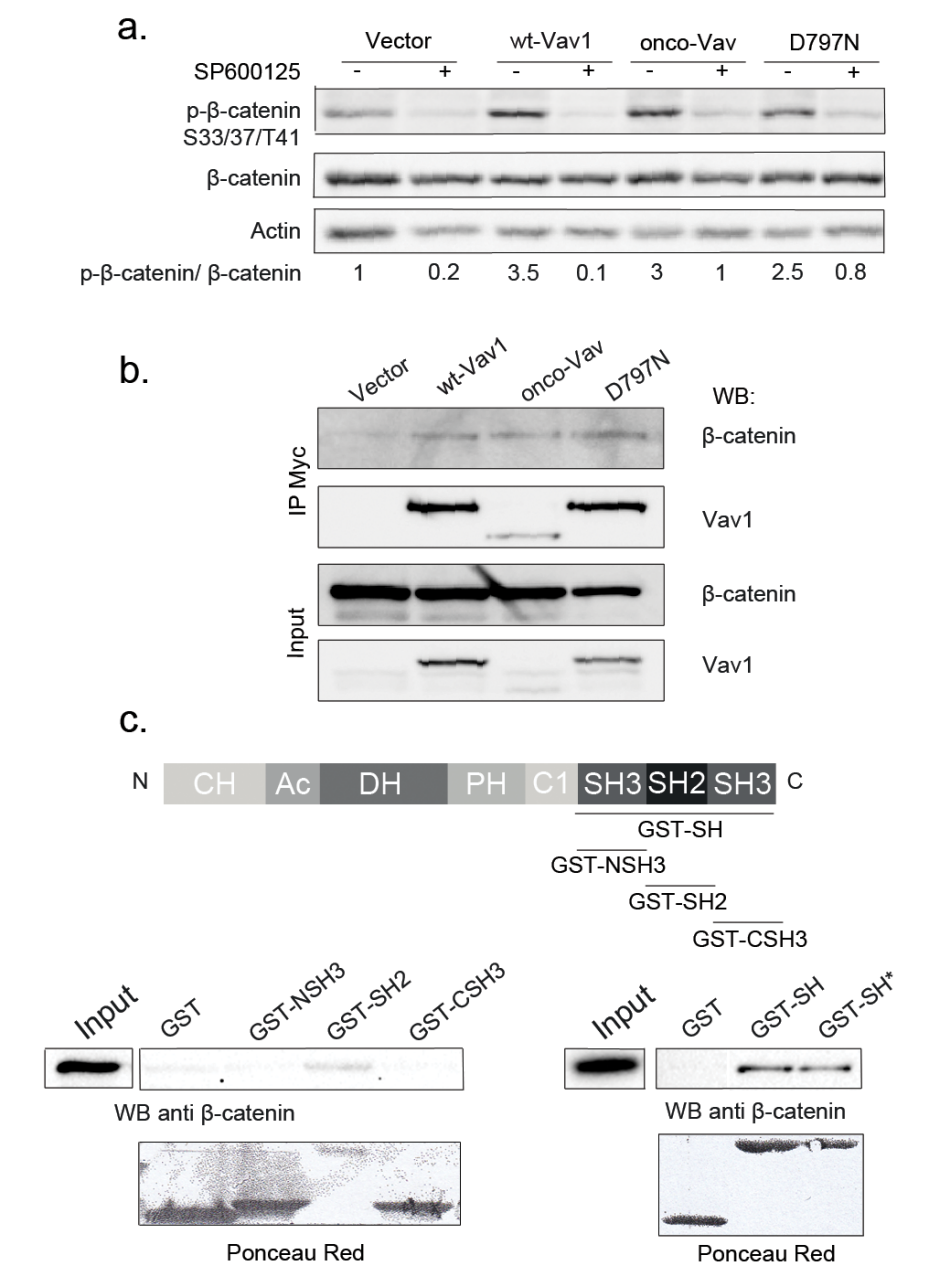
β-catenin is a molecular target of Vav/JNK activation a. β-catenin is a target of JNK. Total cell extracts from stable NIH3T3 cell lines cultured in the presence (+) or not (−) of SP600125 were analysed by sequential immunoblotting with the indicated Abs. Fold increase of phospho-β-catenin in cells expressing wt-Vav1, onco-Vav and D797N compared to control vector were normalized to whole β-catenin. b-c. Vav1 and β-catenin are interacting partners. b. Lysates from the indicated stable NIH3T3 cell lines were immunoprecipitated with anti-Myc Tag Ab. Immune complexes (upper panels) or total extracts (lower panels) were analysed by sequential immunoblotting with the indicated Abs. c. GST-fusion proteins as described in the upper panel were incubated with NIH3T3 cell extracts. Interacting β-catenin was determined by immunoblotting. Total cell extract (Input) was used as a control for β-catenin expression and GST fusion proteins were detected using Ponceau red. GST-SH* corresponds to D797N mutation introduced into GST-SH fusion protein.

Next, we analysed downstream targets of the Rac/JNK-mediated pathway that has proven relevant for cell-cell contacts and adherens junctions. Both Rac1 and JNK2 are involved in the dynamic regulation between the signalling activity and the structural function of the stable pools of β-catenin [31, 35-37]. Immunoblot analysis with antibodies recognizing either β-catenin or its triple phosphorylated form (Ser33/37/Thr41) showed higher phosphorylation in cells expressing tranformants or wt-Vav1 as compared to empty vector (Figure 5a). Phosphorylation was not associated with an overall reduction of β-catenin levels as reported for GSK3-mediated phosphorylation [38, 39]. In the presence of the JNK inhibitor, β-catenin phosphorylation was largely inhibited indicating the involvement of this kinase in a post-translational modification that does not trigger β-catenin for degradation (Figure 5a, 40). Thus, we explored a possible interaction between Vav1 and β-catenin. As shown in Figure 5b, β-catenin specifically co-precipitated with all Vav1 proteins. Using GST-fusion proteins containing either all three (GST-SH) or individual (NSH3, SH2 and CSH3) Src homology domains, we further restricted the interaction with β-catenin to the SH2 domain of Vav1. No interaction was observed with the two GST-SH3 fusion proteins. Interestingly, the D797N mutation introduced into the GST-SH fusion protein (GST-SH*) did not affect Vav1 interaction with β-catenin (Figure 5c). Therefore, these results indicate that Vav1 associates with β-catenin in a complex through its SH2 domain.

### Vav1 influences β-catenin phosphorylation and subcellular localization

**Figure 6:**
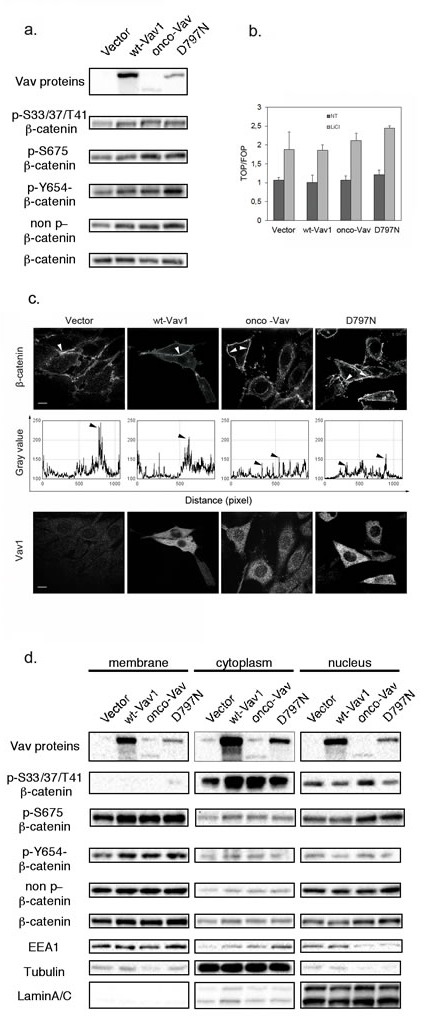
Vav1 influences β-catenin phosphorylation and subcellular localization The indicated cell lines were analyzed using: a and d. Sequential western blotting with the indicated Abs for total cell extracts (a) or after subcellular fractionation between membrane, cytoplasmic and nuclear fractions (d). b. TCF/Lef-luciferase reporter activation was evaluated in 293T cells 48h after transfection with the indicated constructs and incubation (LiCl, grey bars) or not (NT, black bars) with 30 mM LiCl. Expression was calculated relative to levels detected in control cells. Results are means ±SD of three independent experiments performed in triplicates. c. Immunofluorescence microscopy after immunostaining with Alexa 647-conjugated anti-β-catenin (upper panel) and anti-Myc tag Ab followed by Alexa 488-conjugated secondary Ab (Vav, lower panel). Fluorescence intensity of β-catenin labelling was analysed all along the membrane using the Analyze-Plot profile function (graphs in middle panel). Arrows indicate the peaks of intensity observed in the upper panel.

Since subcellular localization and phosphorylation on particular residues of β-catenin are crucial events that modulate its different functions, we further investigated whether Vav1 proteins could have an impact on these modifications. Western blot analysis with anti pS675 β-catenin, a residue phosphorylated by the JNK kinase [29, 40] confirmed increased phosphorylation in Vav1 expressing cells, and more significantly with the two oncogenic forms. Similar increases of pY654- and non phosphorylated- β-catenin were also observed for all Vav1 expressing cells (Figure 6a). A non specific increase of all phosphorylated forms in these cells was ruled out by using anti pY142-, pS552 and pY489- β-catenin that remained with constant levels in all the transfectants (data not shown).

Since phosphorylation of S675 and the non-phosphorylated β-catenin have been implicated in the balance between membrane function and nuclear translocation of β-catenin, we sought to verify whether expression of Vav1 mutants could impact β-catenin transcriptional activity. Upon transient transfection of a luciferase reporter construct based on β-catenin-TCF/LEF transcriptional function we did not observe significant differences between the various Vav1 constructs in 293T cells. On the other hand, treatment with LiCl, a potent inhibitor of GSK3β−dependent degradation of β-catenin resulted in a substantial and comparable induction of the transcriptional response in all cases (Figure 6b).

Next, we evaluated β-catenin distribution at the cell membrane. As expected, endogenous β-catenin was abundantly localized at the cell membrane in control cells with a clustered accumulation at the sites of adhesion and cell-cell contacts. Importantly, expression of Vav1 proteins resulted in a redistribution of β-catenin all along the membrane with a decreased clustering. This peculiar reduction of the clustering of β-catenin was even more pronounced with the two oncogenic Vav1 proteins. Also, we noticed in several cells a significant cytoplasmic accumulation in association with perinuclear labelling (Figure 6c). These results were confirmed by cellular fractionation followed by western blot analysis. The accumulation of pS33/37/T41-β-catenin was detected in the cytoplasmic fraction with few nuclear translocation in onco-Vav-expressing cells only; while the non-phosphorylated counterpart was consistently distributed between membrane and nucleus for all the cells (Figure 6d). Furthermore, accumulations of pY654-β-catenin at the membrane and of pS675-β-catenin both at the membrane and in the nucleus confirmed the difference seen between the different cell lines in whole cell extract. Conversely, random localization of β-catenin at the membrane in onco-Vav and D797N-expressing cells (Figure 6c) was not associated with changes in the overall amounts of β-catenin in these fractions compared to control cells. Finally, substantial amounts of Vav1 proteins were also observed in the nuclear fraction (Figure 6d).

Thus, these results support an impact of Vav1 expression on certain phosphorylations and on structural function of β-catenin at the cell-cell contacts and adhesive complexes. Notably, a differential phosphorylation by wt-Vav1, and more importantly by the two oncogenic proteins, was observed on residues that are targets of Vav1-dependent pathways.

### Ectopic expression of Vav1 allows the modulation of β-catenin in lung cancer cells

**Figure 7:**
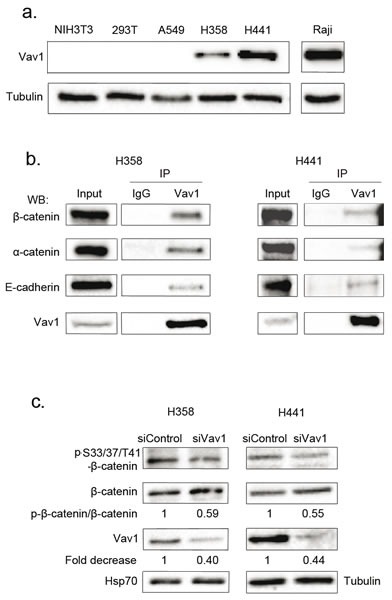
Vav1 interacts with β-catenin in lung epithelial cancer cell lines a. Vav1 protein expression in pulmonary and lymphoid cell lines. Total cell extracts were analysed by sequential immunoblotting with the indicated Abs. b. Total cell extracts from the indicated cells were immunoprecipitated (IP) with anti-Vav1 Ab or control IgG. Immune complexes or total extracts (Input) were analysed by sequential immunoblotting with the indicated Abs. c. Η358 and Η441 cells were transfected with the indicated siRNA and proteins levels assessed 72 hours after transfection. Ratio between phospho-β-catenin and total β-catenin was calculated relative to transfection of control siRNA.

**Figure 8:**
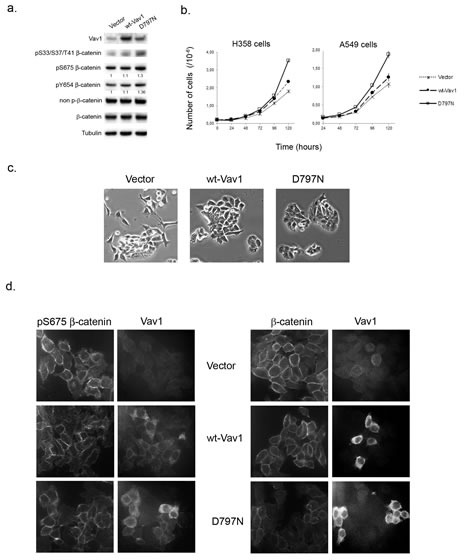
Ectopic expression of Vav1 allows modulation of β-catenin in lung cancer cells a. Total cell extracts from stable H358 cells expressing control vector only, wt-Vav1 or the D797N mutant were analysed by immunoblotting with the indicated Abs. Fold increase of phospho-Y654- and phospho-S675-β-catenin in cells expressing wt-Vav1 and D797N compared to control cells were normalized to total β-catenin. b. Wt-Vav1 and D797N expressing cells proliferation. 2.10^5^ stable H358 cells (left panel) or transiently transfected A549 cells (right panel) expressing the indicated constructs were seeded in 6-wells plate in duplicate and counted at the indicated times. Each value represents the mean of the cell number ± standard deviation from one representative experiment of a total of three. c. Morphology of H358 cells. Stable H358 cells expressing the indicated constructs were cultured below sub-confluence and photographed by phase contrast (magnification. 20x). d. Immunofluorescence analysis of β-catenin in H358 cell lines. The indicated stable H358 cell lines were analysed after anti-pS675β-catenin and Vav1 immunostaining (left panels) or anti- β-catenin and Vav1 immunostaining (right panel) by confocal microscopy.

The ectopic expression of Vav1 is observed in numerous non-hematopoietic cancer cells [10, 12, 13, 41]. As shown in Figure 7a, H358 and H441, two human lung carcinoma epithelial cell lines exhibit substantial amounts of Vav1 proteins compared to a lymphoid cell line (Raji). Our results obtained in fibroblasts prompted us to figure out whether Vav1 interacts with β-catenin in these cells. We confirmed the interaction between endogenous Vav1 and β-catenin in both cell lines. Analyses of Vav1 immunoprecipitates allowed also the specific detection of α-catenin and E-cadherin, two β-catenin well known partners at the cadherin-based adherens junctions (Figure 7b, [40]. Remarkably, the two epithelial cell lines displayed pS33/37/T41β-catenin (Figure 7c). In order to confirm the impact of Vav1 expression on the stability and phosphorylation of β-catenin in these cells, we used RNA interference to reduce endogenous Vav1 expression or alternatively overexpression of wt-Vav1 or D797N. Transfection of Vav1 siRNA into H358 and H441 cells resulted in decreased levels of pS33/37/T41β-catenin as compared to control siRNA while no reduction of the overall β-catenin levels was observed (Figure 7c). This effect of Vav1 was further confirmed with stable overexpression of wt-Vav1 or D797N in H358 cells where higher levels of phosphorylation were detected in such conditions (Figure 8a). Moreover, we confirmed a differential impact of D797N compared to wt-Vav1 on pS33/37/T41-, pS675- and pY654-β-catenin intensities yet the mutant form was expressed at lower levels than wt-Vav1.

Next, the impact of these overexpressions on the proliferation rate and the cell morphology were evaluated. As shown in Figure 8b (left panel) H358 cells expressing D797N mutant exhibited a rapid overgrowth as compared to wt-Vav1 or control vector. Similarly, when transiently transfected with D797N mutant, A549 cells that do not express endogenous Vav1 (Figure 7a), showed as early as 72 hrs post transfection a proliferative advantage as compared to control cells (Figure 8b right panel). Morphological changes were also observed. While H358 cells expressing wt-Vav1 presented with an intermediate shape, showing less protrusions and stretched morphology than vector-transfected cells, D797N appeared with round shaped cells forming multi-layers groups of cells. Finally, immunofluorescence analysis of H358 with anti-pS675 β-catenin Ab indicated a more random localization at the membrane of the phosphorylated protein in wt-Vav1 or D797N cells compared to control cells that kept a punctuated labeling at sites of cell-cell contact, whereas total β-catenin remained unchanged between the three cell lines (Figure 8d).

These results demonstrate that the expression of Vav1 contributes in cancer cells to the phosphorylation at specific residues of β-catenin with an advantage for the D797N mutant that demonstrated in fibroblasts higher GEF activity. These modifications of β-catenin participate to the modulation of its function at cell-cell junctions and to the subsequent alteration of cell shape and growth. Moreover, Vav1 interacts also with β-catenin partners in adherens junctions strongly suggesting a key role in cell-cell adhesion.

## DISCUSSION

This study highlights the essential role of the adaptor region of Vav1 not only as a scaffold domain for immune receptors signalosomes but also as a tranformation potentiator. Using, in Vav1 proto-oncogene, the unique mutation of an aspartic acid residue, specific for the Vav family of proteins, we show that alteration of the C-terminal SH3 domain activates the transforming capacity of the proto-oncogene. A previous study, addressing the role of the CSH3 domain in the transforming capacity of onco-Vav, showed that the D797N substitution did not abrogate the oncogenicity and was considered as unsignificant compared to mutations on conserved residues among SH3 domains [23]. Importantly, our results show that this single mutation, in the full-length Vav1 proto-oncogene, is able to induce activation and consequently oncogenesis.

This gain of function mutation occurs on a charged residue that does not belong to the consensus residues required for the structural integrity of the SH3 domains [23, 42]. It is rather placed in a hinge section available for interaction with negatively charged residues of other domains or proteins. A previous study already showed that the mutation on this residue is not likely to affect the interaction with other partners [23]. We show here that the novel interaction with β-catenin is conserved with the mutant protein. Crystallographic resolution of the full-length molecule has not been reported so far; however, several mutational or biochemical approaches have given insights to the regulation of Vav functions through conformational changes occurring notably upon a stimulation process [9, 21, 24]. Alterations activating the transforming activity have been described for other SH-containing proteins and have been reported in human cancers. For example, mutations of the kinases Src and Abl or of the exchange factor Dbl in domains adjacent to their catalytic site greatly activate their oncogenic potential [43-45]. So far, oncogenicity of Vav1 has been ascribed to an activation of its GEF activity following the deletion of the autoinhibitory loop present at the N-terminus of the protein [6, 21, 46, 47]. This deletion allows further regulation of the GEF activity by phosphorylation of tyrosine residues present within the acidic region [48]. These tyrosine residues are hidden by intra-molecular interactions that prevent the GEF activity [47-49]. Additional intramolecular folding was shown to be important for Vav functions [50]. Recently, an elegant mutational study demonstrated that the C-SH3 domain highly contributes to the formation of the autoinhibited state of the full length Vav1 proto-oncogene [24]. Among others, the D797 residue is mediating such regulatory intramolecular interactions and consequently, the D/N mutation should disrupt such a contact leading to potentiation of the GEF activity as seen in the Rac GTPase assay. This increased GEF activity is also evidenced by enhanced phosphorylated state of the D797N mutant compared to the wt-Vav1.

One key finding of this work is the identification of a new partner for Vav1, β-catenin. The SH2-domain of Vav1 mediates this interaction likely by recognition of phosphorylated tyrosine residues of β-catenin. Multiple phosphorylated Ser/Thr or Tyr residues of β-catenin are involved in the regulation of the protein. In particular, several sequence motives contribute to conformational changes that lead to signalling functions or recognition by the proteasomal machinery for its degradation [40]. Our results show that Vav1 expression activates the Rac/JNK pathway in fibroblasts and, its oncogenic mutations potentiate this activity. Activation of JNK induces phosphorylation and stabilization of β-catenin at sites also targeted by GSK-3β, an important component of the degradation complex together with Axin and APC [29, 40, 51]. In this work we show that Vav1/JNK-dependent phosphorylation on both S675 and S33/37/T41 rather stabilizes β-catenin and its phosphorylated forms in different cellular compartments. Similar effects of JNK have been described in keratinocytes and in colon cancer cells together with an inhibitory role on the formation of clustered adhesive complexes with α-catenin and E-cadherin [29, 36, 37]. Expression of the oncogenic mutants in fibroblasts leads to redistribution of β-catenin along the membrane with a reduction of adhesion clusters and some translocation to the cytosol. These results suggest that Vav1 and its oncogenic forms can serve as adaptors and potentiators bridging JNK kinase with effector β-catenin that will modify cohesion at the cell-cell adhesion sites. We showed an association of Vav1 within the adherens junction complexes involving α-catenin and E-cadherin in epithelial cancer cell lines. In these cells we also evidence a potentiator role for Vav1, and moreover for the activated D797N mutant, in the phosphorylation of β-catenin at specific residues and some redistribution of β-catenin at the membrane along with modification of the cell shape. Interestingly, a recent work highlighted a role for Vav1 in Epithelial Mesenchymal Transition (EMT) in ovarian cancer [52] and it has been demonstrated that β-catenin might participate in this process [53]. Further work will figure out how Vav1 might contribute to the modulation of the assembly of adherens junctions. Besides Vav1, Vav2, a more ubiquitously expressed member of the family of adaptors, also interacts with components of the adhesion complex, e.g. through p120-catenin in SW-480 colon cancer cells [54]. Particularly, Vav2 interacts with Ser-phosphorylated, Tyr-dephosphorylated cytosolic p120-catenin that has been released from the E-cadherin-Wnt receptor complex at the cell membrane upon Wnt stimulation [30].

In other cellular processes, such as osteoblastogenesis, or in colon cancer cells, a Rac1/JNK2-dependent phosphorylation of β-catenin was described at sites that regulate its nuclear accumulation and signalling function [31]. The transport of β-catenin to the nucleus represents an important regulation node for the balance between its adhesive and signalling properties [55]. Our biochemical fractionation and immunofluorescence analysis in fibroblasts showed an increased perinuclear β-catenin labelling in the presence of the transforming forms of Vav1 but a rather weak translocation to the nucleus for the pS33/37/T41 stabilized form and a more evident nuclear accumulation for pS675 β-catenin. In our experimental settings, in the presence of Vav1 oncogenic forms, we observed a Vav1-JNK dependent activation of c-Jun but we did not observe the transcriptional activation of β-catenin associated with its stabilization. However, we observed that the D797N mutant confers a growth advantage when overexpressed in lung cancer cells. We cannot rule out a putative Vav1-dependent cooperation between AP1 and β-catenin to regulate tumour progression and further work is needed to address this point [56, 57].

In light with the recent findings on Vav1 as a central regulator of invadopodia [15], our study reveals a new aspect of Vav1 functions when its deregulated expression favors the interaction with additional partners that leads to the alteration of signalling pathways involved in developmental processes or cell contact inhibition and that can ultimately result in growth advantage or tumorigenesis. Additionally, our results strengthen the importance of activating single point mutations in the C-SH3 domain that abrogate Vav1 autoinhibition, potentiate the GEF activity and consequently facilitate tumour progression. Since mutation on the adaptor protein can affect cell-contact inhibition, Vav seems an interesting target to modulate signalling during tumorigenesis.

## METHODS

### Mammalian expression plasmids and GST fusion proteins

The pEF-neo expression plasmids encoding Myc-tag human wt-Vav1 has been previously described [58]. Plasmids containing Δ1−66 onco-Vav and A789N, D797N and G830V mutants, were subcloned in pEF-neo expression vectors. Wt-vav1 and D797N mutant were also subcloned in pcDNA. pRK5 expression plasmids encoding Myc-tagged Rac1(17N) and RhoA(19N) were kindly provided by Dr. O. Dorseuil (Institut Cochin, U1016 INSERM, Paris, France). PCR amplified N-terminal Src homology (NSH3), SH2 and CSH3 or whole Src (SH) domains of Vav were subcloned in pGEX-4T-1 or pGEX-4T-3 (Pharmacia Biotech Inc.)

### Antibodies

Antibodies for Myc-Tag, Vav1, JNK, p-JNK, p38, p-p38, lamin A/C, p-βcatenin S33/37/T41, Hsp70, P-c-Jun, c-jun, pY654-β-catenin, pS675- β-catenin, no-P- β-catenin and Alexa 647-conjugated anti-β-catenin were obtained from Cell Signalling Technology, Anti-β-catenin, anti-E-cadherin, EEA1 Abs and anti-IgG control isotype were purchased from Becton Dickinson (BD), anti-α-catenin and anti-Tyr174 Vav1 from Santa-Cruz, and anti-β actin or anti-tubulin from Sigma.

### Cell culture, transfection and focus formation assay

NIH3T3 cells were cultured in DMEM supplemented with 10% Newborn Calf Serum (NCS; Invitrogen and PAA). For the focus formation assays, cells were cultured for 12-14 days after transfection with Lipofectamine and Reagent Plus (Invitrogen); medium was replaced every 2 or 3 days and was supplemented or not with JNK (SP600125) or P38 (SB203580) kinases inhibitors (Calbiochem). Foci were scored after Giemsa staining. Stable NIH3T3 cell lines expressing the various Vav proteins and vector control were selected in the presence of 1 mg/ml neomycin after transfection. Neomycin-resistant colonies were scored after 10-12 days of selection.

H358, H441 and A549 cell lines were maintained in RPMI and DMEM respectively, supplemented with 10% Foetal Bovine Serum (FBS; PAA). H358 and A549 cell lines were transfected with pcDNA constructs by using the Amaxa or JetPrime (Polyplus) following recommendation.

SiRNA control or targeting Vav1 were purchased from Dharmacon and transfected using JetPRIME reagent (Polyplus).

### Anchorage-independent growth assay

NIH3T3 cells were cultured in DMEM-10% NCS containing 0,4% agar over a layer of DMEM-10% NCS containing 0.8% agar during 21 days. Growth medium was added every other week.

### Tumorigenic assay in Nude mice

Stable NIH3T3 cells lines were injected subcutaneously in Nu/Nu mice (1.10^6^ cells). Development of tumours was monitored weekly.

### Protein stability assay using cycloheximide

4.10^5^ cells were seeded in 6-well dishes 24h prior to treatment with 100 μM cycloheximide. Cells were harvested at different time points after drug addition (2-8 hours). Stability of the Vav mutant proteins was evaluated by western blot.

### Immunoprecipitation and western blot analyses

Protein extracts were prepared in Triton lysis buffer (50mM Tris-HCL [pH 7.5], 150mM NaCl, 10% glycerol, 1mM EDTA, 0.1% Triton-X100) containing protease inhibitors (10 μg/ml aprotinin, 5 μg/ml leupeptin, 5 μg/ml pepstatin, 1mM phenylmethyl sulfonyl fluoride (PMSF), 1mM DTT, 25mM β-glycerophosphate, 1mM sodium fluoride and 1mM Na_3_VO_4_). Proteins were separated by SDS-PAGE transferred to Hybond membrane (Amersham) and probed with appropriate primary and HRP-conjugated secondary (Sigma) Abs. Quantitation was performed using Image Lab 4.10 (Bio-Rad). For immunoprecipitation, Triton-X100 was replaced by 1% NP-40. After preclearing, cell extracts were incubated overnight with the indicated Abs or agarose beads-conjugated anti-Myc-tag Ab (Sigma). Beads were further washed with the lysis buffer and precipitates were eluted in 4X Laemmli buffer prior to immunoblotting.

### Subcellular Fractionation

Nuclear, membrane and cytoplasmic fractions were prepared using the Subcellular Protein Fractionation Kit for Cultured Cells (Thermo Scientific) as per manufacturer's instruction. Protein concentration was quantitated using the BCA protein assay kit (Pierce).

### GST pull-down assay

Expression of the Glutathione S-transferase (GST) fusion proteins was induced with 500μM isopropyl-β-thiogalactopyranoside (IPTG). Fusion proteins were purified from bacterial lysates using gluthatione-Sepharose 4B beads (GE Healthcare). Proteins extracts from NIH3T3 stable cell lines were incubated 2 hours with fusion proteins bound to gluthatione-Sepharose 4B beads. Precipitates were recovered as described for immunoprecipitation assay and analysed by immunoblotting.

### Luciferase reporter assay

293T cells were seeded in 12-well dishes 24h prior to transfection using JetPrime (Polyplus) with Vav constructs, Top-firefly or Fop-firefly negative control luciferase plasmids (Millipore) and Renilla luciferase plasmid under the control of SV40 promoter, pRL(Promega). 48h after transfection, cell lysis was performed using the Passive Lysis buffer followed by the Dual luciferase assay (Promega). Firefly and Renilla luciferase activities were monitored following manufacturer's instructions. LiCl (30mM) treatment was applied 3 hours before lysis. Top-firefly luciferase values were normalized to the respective Renilla and Fop-luciferase values for each treatment.

### Immunofluorescence

Cells were fixed in paraformaldehyde (4%) and permeabilised with 0.25% Triton-X100 in PBS. Cells were stained with the indicated Abs. Actin filaments were visualized with Alexa 546- or Alexa 647-conjugated-phalloidin. Confocal images were acquired under a BD Pathway 855 Bioimaging System with a 40x objectif and further analysed using AttoVision and Image J softwares.

### Rac1 Activation Assay

Rac1 activation assays were performed using the Active Rac1 Pull-Down and Detection Kit. The assay consists on a GST-pull down experiment with the p21-binding domain of PAK1 that selectively precipitates active Rac1 from whole cell extracts. Active Rac1 is quantified by immunoblot analysis using anti-Rac1 monoclonal Ab (Thermo Scientific Pierce).

### Statistical analysis

Statistical significance was assessed using impaired Student's t test (Prism5.0, Graph Pad Software). P values <0.05 were considered significant with the following degrees: *p<0.05; **p<0.01; ***p<0.001.

## SUPPLEMENTARY MATERIAL, FIGURES



## The abbreviations used are

SH: Src homology domain
Ab: Antibody
GEF: GDP/GTP exchange factor
wt: wild type
dn: dominant-negative
JNK: c-jun NH2-terminal kinase
GST: Glutathione S-transferase
PMSF: phenylmethyl sulfonyl fluoride
