# Supplementary material for: Mutation of Vav1 adaptor region reveals a new oncogenic activation
Source: Oncotarget. 2014 Oct 24;6(4):2524–38. doi: 10.18632/oncotarget.2629 (PMC4385868; doi:10.18632/oncotarget.2629)
Supplement: Supplementary file 1 [file oncotarget-06-2524-s001.pdf]

Mutation of Vav1 adaptor region reveals a new oncogenic activation

Supplementary Material

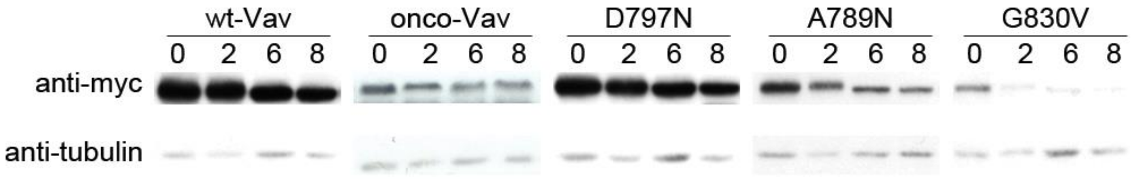

Figure S1: Stability of the Vav mutant proteins. Stable NIH3T3 cell lines expressing the various constructs or control vector were treated with cycloheximide for the indicated periods of time and lysed. Cell extracts (equivalent to 10^5 cells) were analysed for the Vav proteins content after immunoblotting with anti-Myc Ab and reprobed with anti-β tubulin for loading control.

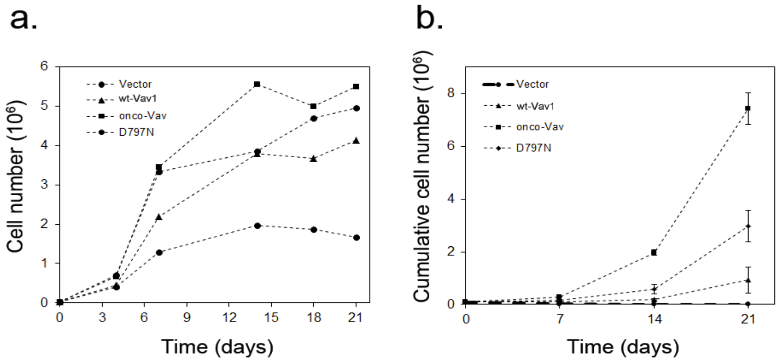

Figure S2: Growth characteristics of Vav1-transformed NIH3T3 cells. Growth capacities of NIH3T3 stable cell lines expressing Wt-, onco–Vav or D797N. Exponentially growing cells were seeded in 6-well plates in duplicates. **a**. 2.5 x 10^4 cells /well in regular medium. Cells were counted at the indicated times. **b**. 1 x 10^5 cells /well; one day after seeding, medium was replaced by 0.5% NCS containing medium. Cells were counted and re-seeded at 10^5 cells /well at the indicated times. Each value represents the mean of the cell number (a) or the cumulative number of expanded cells (b) ± standard deviation from one representative of three independent experiments.

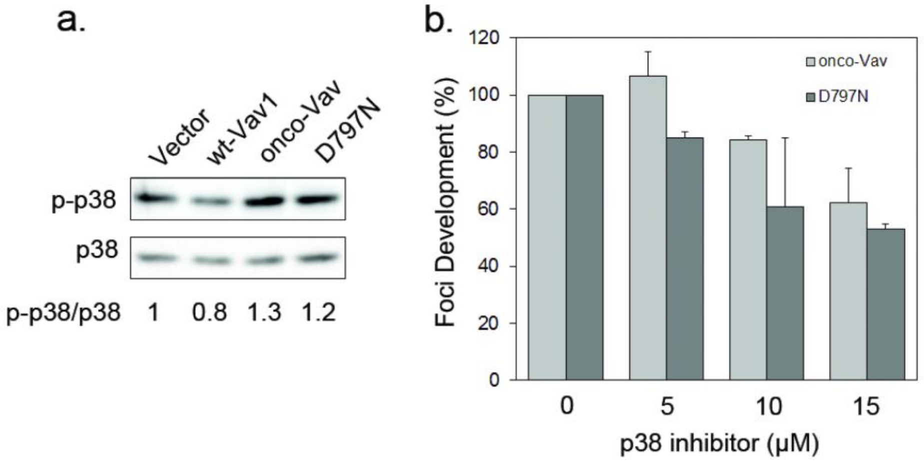

Figure S3: P38 activation is not required for D797N and onco-Vav transformation. a. p38 SAPK phosphorylation analysis. Protein extracts from the indicated stable NIH3T3 cell lines were analysed by sequential immunoblotting with anti-phospho-p38 (Thr180/Tyr182) and anti-p38 Abs. Fold increase of phospho-p38 in cells expressing wt-Vav1, onco-Vav and D797N compared to control vector is normalized to whole p38 expression. **b**. p38 activity and transformation. Focus assays were performed with NIH3T3 cells transfected with the indicated constructs in the presence (5, 10 and 15 μM) or not (0) of SB203580. Foci formation is calculated relative to untreated cells (100%). Results are means ± SD of 3 independent experiments performed in duplicate.
